# The fatty acid 2-hydroxylase CsSCS7 is a key hyphal growth factor and potential control target in *Colletotrichum siamense*

**DOI:** 10.1128/mbio.02015-23

**Published:** 2024-01-10

**Authors:** Yitao Xi, Xiping Long, Miao Song, Yu Liu, Jingting Yan, Yanyun Lv, Hong Yang, Yu Zhang, Weiguo Miao, Chunhua Lin

**Affiliations:** 1 Sanya Institute of Breeding and Mutiplication, Key Laboratory of Green Prevention and Control of Tropical Plant Diseases and Pest (Ministry of Education)/School of Tropical Agriculture and Forestry, Hainan University, Haikou, China; 2 Rubber Research Institute, Chinese Academy of Tropical Agricultural Science, Haikou, China; Duke University School of Medicine, Durham, North Carolina, USA

**Keywords:** *Colletotrichum siamense*, fatty acid hydroxylase CsSCS7, hyphal growth factor, control target

## Abstract

SCS7 is a fatty acid 2-hydroxylase required for the synthesis of inositol phosphorylceramide but is not essential for normal growth in *Saccharomyces cerevisiae*. Here, we demonstrate that the *Colletotrichum siamense* SCS7 homolog CsSCS7 plays a key role in hyphal growth. The *CsSCS7* deletion mutant showed strong hyphal growth inhibition, small conidia, and marginally reduced sporulation and also resulted in a sharp reduction in the full virulence and increasing the fungicide sensitivity. The three protein domains (a cytochrome b5 domain, a transmembrane domain, and a hydroxylase domain) are important to CsSCS7 protein function in hyphal growth. The fatty acid assay results revealed that the *CsSCS7* gene is important for balancing the contents of multiple mid-long- and short-chain fatty acids. Additionally, the retarded growth and virulence of *C. siamense* Δ*CsSCS7* can be recovered partly by the reintroduction of homologous sequences from *Magnaporthe oryzae* and *Fusarium graminearum* but not *SCS7* of *S. cerevisiae*. In addition, the spraying of *C. siamense* with naked *CsSCS7*-double-stranded RNA (dsRNAs), which leads to RNAi, increases the inhibition of hyphal growth and slightly decreases disease lesions. Then, we used nano material Mg-Al-layered double hydroxide as carriers to deliver dsRNA, which significantly enhanced the control effect of dsRNA, and the lesion area was obviously reduced. These data indicated that *CsSCS7* is an important factor for hyphal growth and affects virulence and may be a potential control target in *C. siamense* and even in filamentous plant pathogenic fungi.

**IMPORTANCE:**

CsSCS7, which is homologous to yeast fatty acid 2-hydroxylase SCS7, was confirmed to play a key role in the hyphal growth of *Colletotrichum siamense* and affect its virulence. The CsSCS7 gene is involved in the synthesis and metabolism of fatty acids. Homologs from the filamentous fungi *Magnaporthe oryzae* and *Fusarium graminearum* can recover the retarded growth and virulence of *C. siamense* ΔCsSCS7. The spraying of double-stranded RNAs targeting CsSCS7 can inhibit hyphal growth and reduce the disease lesion area to some extent. After using nano material Mg-Al layered double hydroxide as carrier, the inhibition rates were significantly increased. We demonstrated that CsSCS7 is an important factor for hyphal growth and affects virulence and may be a potential control target in *C. siamense* and even in filamentous plant pathogenic fungi.

## INTRODUCTION

Eukaryotic filamentous fungi, including *Magnaporthe oryzae*, *Fusarium graminearum*, and *Colletotrichum* spp., are among the dominant causal agents of plant diseases that can cause extensive yield losses in major agricultural crops worldwide (1). One of the dominant cell types of filamentous fungi is hyphae, which is important for fungal invasion and colonization of substrates and searching for mating partners or host organisms (2). The key factors for hyphal growth may be a potential target in the control of plant pathogenic filamentous fungal diseases.

Sphingolipids are ubiquitous and abundant components of eukaryotic plasma membranes and play multiple important roles in signal transduction, intracellular trafficking, apoptosis, cell differentiation, and other processes (3, 4). Fungal sphingolipids play a pivotal role in growth, morphogenesis, and virulence and have potential as targets in future antifungal therapies (5, 6). Fungi possess unique enzymes involved in the synthesis of fungal sphingolipids, which can produce lipids that are structurally different from their mammalian counterparts (5). A deeper knowledge of sphingolipid metabolism is key to the development of new drugs. Most of the information available on fungal sphingolipid metabolism comes from the model organism *Saccharomyces cerevisiae* (7, 8). Some details of the fungal sphingolipid metabolism of several filamentous fungi, such as *Aspergillus*, *Cryptococcus*, and *Histoplasma*, have also been described (6, 9–13). Fungal glycosphingolipids are clustered along with sterols in specialized membrane microdomains termed lipid rafts, which play a crucial role in the establishment of fungal cell polarity (14). Lipid rafts have been observed in hyphal tips of *A. nidulans*, and the synthesis and localization of sphingolipids at these active growth sites appear to be relevant for its differentiation because the disruption of sphingolipid production by myriocin treatment impairs establishment of the cell polarity axis in spores and prevents normal hyphal branching in germlings (10, 15). However, the effects of the synthesis and differentiation of sphingolipids on the growth of filamentous fungal hyphae remain unclear.

The structural diversity of sphingolipids has been extensively studied, and the studies have focused on their tree building blocks, the headgroup, the acyl chain, and the sphingoid base (16). Hydroxylation of the sphingoid base can add structural diversity that appears to be important for the physiological function of sphingolipids (4). Hydroxylation can occur at C-2, C-3, and ω-C of the acyl residue and at C-4 of the sphingoid base and is catalyzed by fatty acid hydroxylase (17, 18). In *S. cerevisiae*, Scs7p is required for hydroxylation of the very long-chain fatty acid at the C-2 position, and Sur2p is needed for hydroxylation of C-4 of the sphingoid moiety of ceramide, but neither SCS7 nor SUR2 are essential for unicellular yeast growth (19). However, sphingolipid C4 hydroxylase BasA (Sur2 homolog) is required for phytosphingosine biosynthesis and essential for viability in the filamentous fungi *A. nidulans* (20). Do sphingolipid hydroxylases have different effects on the hyphal growth of unicellular and filamentous fungi? The functional role of SCS7 orthologs in filamentous fungi of plant pathogens has not been elucidated to date.

*Colletotrichum* spp. are members of an important genus of plant pathogenic filamentous fungi (1). *C. siamense* is reportedly one of the major pathogenic species of many tropical or subtropical crops, including rubber trees (21). Our research group previously screened and identified the fatty acid hydroxylase CsSCS7, which exhibit 51% identity to *S. cerevisiae* SCS7, as the interacting protein of the core member CsPbs2 of the HOG MAPK in *C. siamense* (22). In this study, the function of *CsSCS7* in *C. siamense* was characterized. Our findings revealed that *CsSCS7* is an important factor for hyphal growth and virulence in *C. siamense*. The fatty acid content of the *CsSCS7* gene null mutant and the wild-type strain revealed that *CsSCS7* is important for balancing the content of multiple fatty acids. The homologs of SCS7 in *M. oryzae* and *F. graminearum* but not that of *S. cerevisiae* induce recovery of the phenotype in terms of growth and virulence. Furthermore, the spraying of *C. siamense* with double-stranded RNAs (dsRNAs) targeting *CsSCS7*, which caused RNAi, increased the inhibition of hyphal growth and decreased disease lesions. Based on these results, we conclude that the *CsSCS7* gene is conserved and plays a critical role in hyphal growth and virulence in filamentous fungi and can thus serve as a potential target in the control of fungal diseases.

## RESULTS

### Identification of the fatty acid 2-hydroxylase CsSCS7 in *C. siamense*

**Fig 1:**
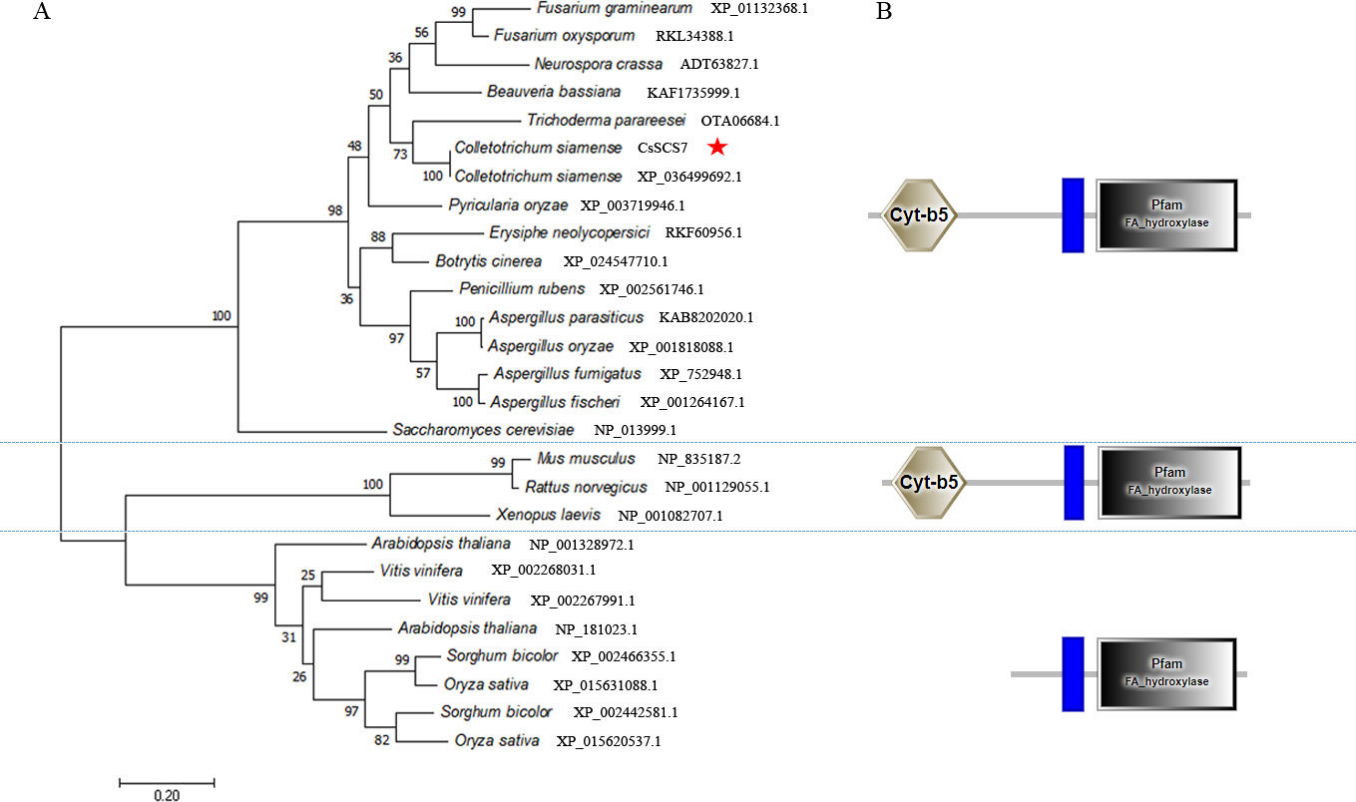
Phylogenetic analysis and protein domains of CsSCS7. (**A**) Phylogenetic analysis of CsSCS7 and its orthologs in other fungal species, plants, and mammals. A phylogenetic tree was constructed with MEGA 7.0 using the maximum-likelihood method. The CsSCS7 protein amino acid sequence in this study is indicated with a red star. (**B**) SMART analysis of the CsSCS7 orthologs from fungi, mammals, and plants.

Our previous study showed that a fatty acid hydroxylase is the CsPbs2-interacting protein by the Y2H system and pull-down method (22). To characterize the function of this gene, the whole coding sequence was obtained from the *C. siamense* HN08 strain by PCR and RT-PCR. Sequence analysis revealed a DNA size of 1,271 bp and a cDNA size of 1,215 bp with one intron. The gene encoded 404 amino acids with a Cyt b5 domain, a transmembrane domain, and a hydroxylase domain (Fig. 1). The gene was annotated in GenBank as a fatty acid 2-hydroxylase and was proven to be homologous to *S. cerevisiae* SCS7. We named the gene *CsSCS7* in *C. siamense* and deposited the sequence into GenBank (accession no. MT943621). The phylogenetic tree of CsSCS7 and its orthologs in other fungi, mammals (FA2H), and plants (FAH1 and FAH2) was constructed. This tree revealed that there was only one SCS7 homolog with a Cyt b5 domain, a transmembrane domain, and a hydroxylase domain in all fungi or a FA2H in mammals, whereas there were two FAHs without a Cyt b5 domain in plants, as previously reported (23).

### *CsSCS7* is an essential factor for hyphal and conidial growth of *C. siamense*

To characterize the biological function of *CsSCS7* in *C. siamense*, we used functional genetic techniques to generate a *CsSCS7* gene deletion mutant using the homologous recombination method (Fig. S1). A total of 73 transformants were obtained based on resistance to sulfonylurea. Among these transformants, a ∆*CsSCS7* mutant was confirmed as a *CsSCS7* gene deletion mutant by PCR amplification, sequencing, and southern blot analysis. The coding sequence of the *CsSCS7* gene could not be amplified, and the fragments upstream of the *CsSCS7* and *ILV1* 5′-terminal sequences and downstream of the *CsSCS7* and *ILV1* 3′-terminal sequences could be amplified. The PCR products amplified from mutant and HN08 genomic DNA using the primers CsSCS7-Ou-F/CsSCS7-Ou-R were sequenced, which yielded a 5,293-bp fragment from ∆*CsSCS7* and a 3,747-bp fragment from HN08. This result indicated that the CsSCS7 coding sequence was replaced by the 2,817-bp fragment of the *ILV1* gene. Southern blot analysis showed that the ∆*CsSCS7* mutant contained only one copy of the *ILV1* gene (Fig. S1C). Therefore, ∆*CsSCS7* was indeed a *CsSCS7* gene deletion mutant. The complementary mutant *ΔCsSCS7/CsSCS7* was also constructed by reintroducing the pXY203-CsSCS7 plasmid (containing the *hph* gene, trpC promoter, and whole coding sequence of the *CsSCS7* gene) into the ∆*CsSCS7* mutant.

**Fig 2:**
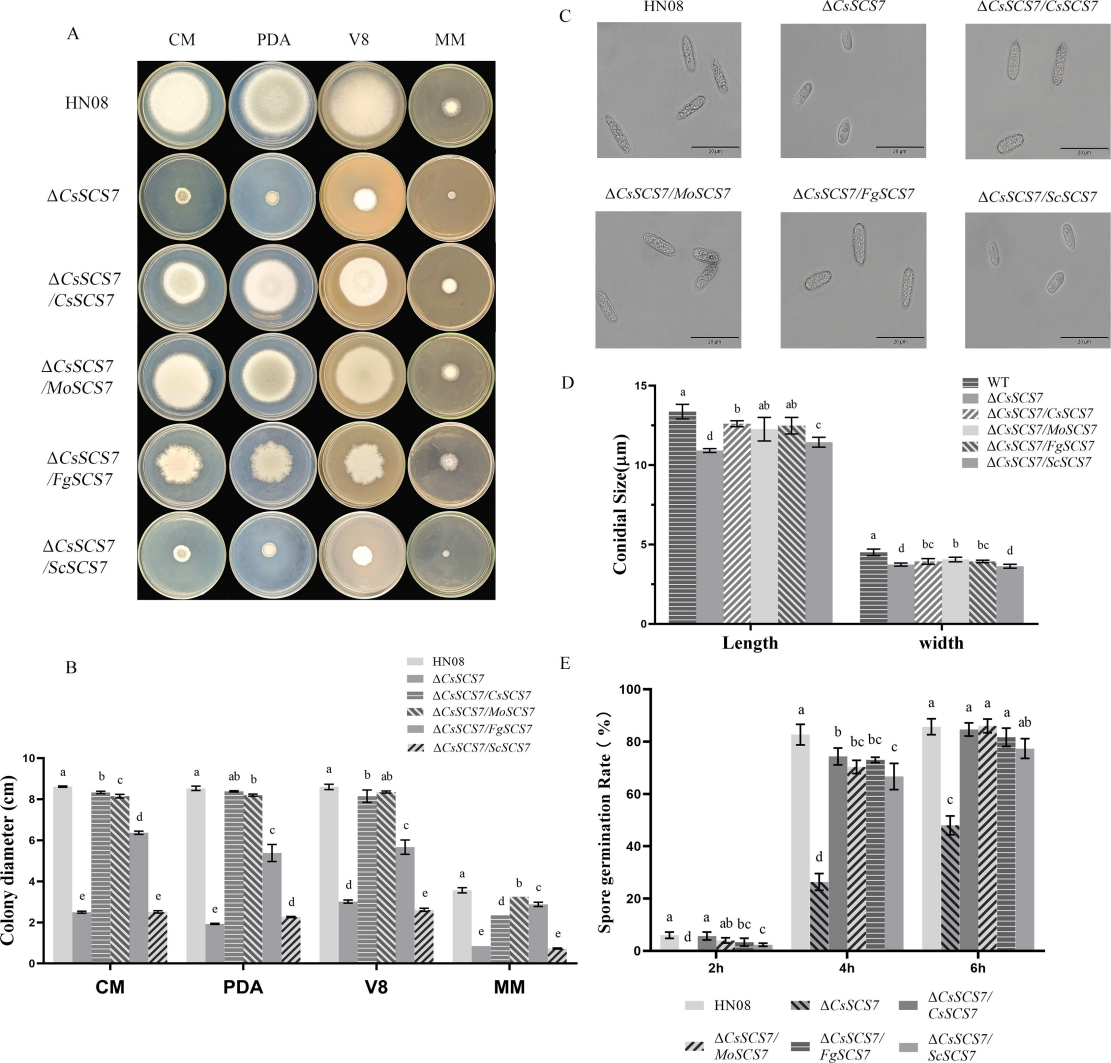
Comparison of colony and conidial characterization of the tested strains. (**A**) Colony morphology and (**B**) colony diameter of the tested strains grown on four types of plates for 7 days. (**C**) Morphology and (**D**) conidial size of the tested strains. (**E**) Spore germination rates of the tested strains. Different letters indicate an extremely significant difference (*P* < 0.01) (one-way analysis of variance [ANOVA] and Duncan’s test), and the error bars represent the standard deviations.

Interestingly, the hyphal growth of Δ*CsSCS7* was markedly reduced (Fig. 2; Table S2). Therefore, we first measured the colony diameter of wild-type HN08, Δ*CsSCS7*, and Δ*CsSCS7/CsSCS7* on different plates. The Δ*CsSCS7* colony diameters after culture on CM, PDA, V8, and MM plates for 7 days were 29.07%, 22.63%, 35.00%, and 23.88% of that of the wild-type strain (data shown in Table S2; Fig. 2), respectively. The complementary strain Δ*CsSCS7/CsSCS7* almost restored the colony growth, and its colony diameters were 96.51%, 98.24%, 94.77%, and 66.01% of that of the wild-type strain, respectively. The results revealed that *CsSCS7* is a key gene for *C. siamense* hyphal growth.

The conidia sizes and spore germination rates were measured and are shown in Table S3; Fig. 2. The conidia size of the ∆*CsSCS7* mutant was (10.91 ± 0.58) × (3.73 ± 0.19) µm, and those of the wild-type and ∆*CsSCS7/CsSCS7* were (13.36 ± 1.98) × (4.50 ± 0.78) µm and (12.40 ± 0.95) × (3.93 ± 0.45) µm (Fig. 2C and D; Table S3), respectively. The data showed that the conidia of ∆*CsSCS7* were significantly small, with a length and width equal to 81.67% and 82.89% of that of wild-type, respectively. The rate of spore germination was measured 2–6 h after inoculation (hai) (Fig. 2E; Table S2). Observations showed that ∆*CsSCS7* conidia did not germinate, but 6.00% of that of the wild-type strain could germinate at 2 hai. At 6 hai, the spore germination rate of ∆*CsSCS7* was only 48.00%, whereas the spore germination rates of the wild-type and ∆*CsSCS7/CsSCS7* strains were as high as 87.00% and 84.67%, respectively. These data indicated that the *CsSCS7* gene had a positive effect on the conidial growth and spore germination of *C. siamense*.

### The *CsSCS7* gene deletion extremely reduces the full virulence of *C. siamense*

**Fig 3:**
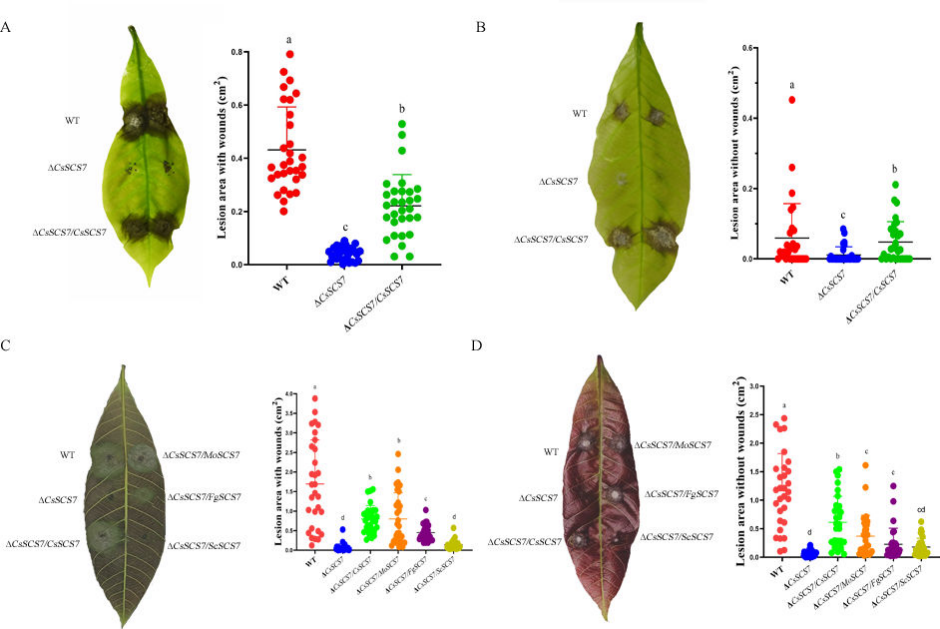
Pathogenicity assays of the strains tested in this study. (**A**) Disease lesions and dot plot of the lesion area of the wild-type strain HN08, Δ*CsSCS7* strain, and complementary strain Δ*CsSCS7/CsSCS7* on rubber tree leaves 3 days after inoculation with wounding. (**B**) Disease lesions and dot plot of the lesion area of the three tested strains on light-green rubber tree leaves 3 days after inoculation without wounding. (**C**) Disease lesions and dot plot of six tested strains on bronzing leaves 3 days after inoculation with wounding. (**D**) Disease lesions and dot plot of six tested strains on bronzing leaves 3 days after inoculation without wounding. Different letters indicate significant differences at *P* < 0.01 according to one-way ANOVA and Duncan’s test; the error bars show the standard deviations.

To test pathogenicity, conidial suspensions (10^5^ conidia/mL) of wild-type HN08, Δ*CsSCS7*, and Δ*CsSCS7/CsSCS7* were inoculated on healthy rubber tree leaves with or without wounding. The disease lesion area and disease incidence were measured 3 days after inoculation (Fig. 3A and B). On the wounded leaves, all three tested strains could infect the light-green leaves and cause symptoms, and the disease incidence was 100%. However, the minimally diseased area caused by Δ*CsSCS7* (mean of 0.04 ± 0.02 cm^2^) was significantly smaller than that caused by wild-type HN08 (mean of 0.43 ± 0.15 cm^2^), and Δ*CsSCS7/CsSCS7* could partly restore the virulence (with a diseased area of 0.22 ± 0.12 cm^2^). On the unwounded leaves, both the disease incidence and lesion area were affected by deletion of the *CsSCS7* gene. The disease incidences of the wild-type HN08, Δ*CsSCS7,* and Δ*CsSCS7/CsSCS7* strains were 63.3%, 33.3%, and 66.6%, respectively. The mean diseased areas caused by HN08, Δ*CsSCS7,* and Δ*CsSCS7/CsSCS7* were 0.06 ± 0.25 cm^2^, 0.01 ± 0.04 cm^2^, and 0.05 ± 0.02 cm^2^, respectively. These results indicated that *CsSCS7* gene deletion attenuated the virulence of *C. siamense* by reducing its ability to penetrate the host surface and the rate of lesion expansion.

### *CsSCS7* gene deletion increases the sensitivity of *C. siamense* to fungicides

**Fig 4:**
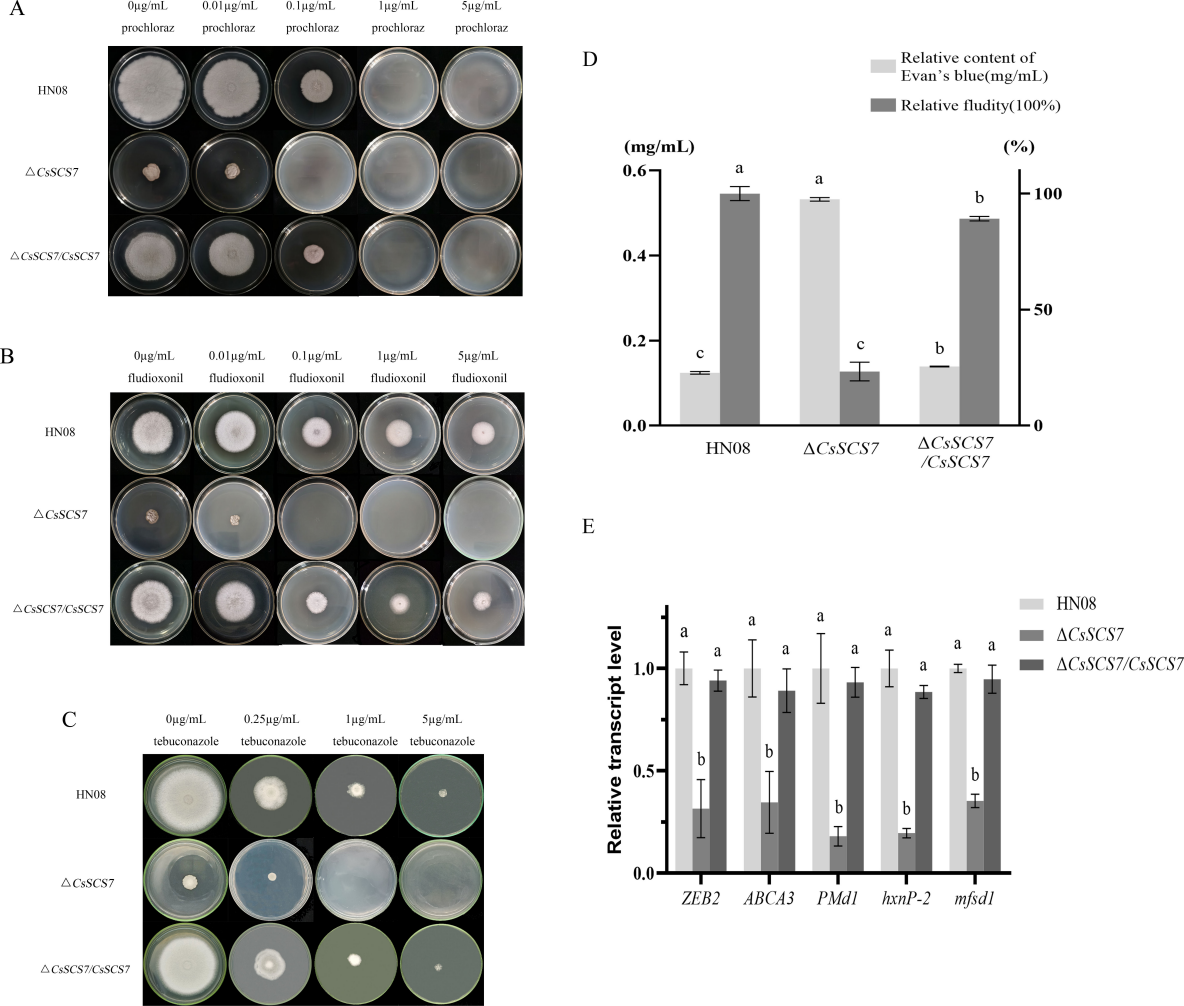
Fungicide sensitivity, the plasma membrane permeability, and gene expression of HN08, ∆*CsSCS7*, and ∆*CsSCS7/CsSCS7*. (**A**) Mycelial growth of tested strains on CM containing prochloraz. (**B**) Mycelial growth of tested strains on CM containing fludioxonil. (**C**) Mycelial growth of tested strains on CM containing tebuconazole. (**D**) Evan’s blue content in HN08, ∆*CsSCS7*, and ∆*CsSCS7/CsSCS7*. The experiment was repeated three times. Different letters indicate significant differences at *P* < 0.01 according to one-way ANOVA and Duncan’s test; the error bars show the standard deviations. (**E**) The expression of drug efflux pump-related genes on three tested strains. *ZEB2* (XP_036488279.1), *ABCA3* (XM_036646271.1), and *PMd1* (XM_036645902.1) from ATP-binding cassette transporters, *hxnp-3* (XP_036497635.1) and *mfsd1* (XP_036489548.1) from the major facilitator superfamily (MFS). ATP-binding cassette transporters and major facilitator superfamily are the main gene families of drug efflux pumps.

To evaluate whether *CsSCS7* is involved in the regulation of fungicide sensitivity in *C. siamense*, the mycelial growth rates of the individual strains on CM plates containing different concentrations of fludioxonil, tebuconazole, and prochloraz were evaluated (Fig. 4). As shown in Fig. 4, 1 μg/mL prochloraz was lethal to the wild-type strain HN08 and the complementary strain ∆*CsSCS7/CsSCS7*, but 0.1 µg/mL prochloraz was lethal to Δ*CsSCS7*. Fludioxonil and tebuconazole (5 µg/mL) were not lethal to HN08 and ∆*CsSCS7/CsSCS7*, but 0.1 µg/mL fludioxonil and 1 µg/mL tebuconazole were lethal to ∆*CsSCS7*. These findings indicated that loss of the *CsSCS7* gene increased the sensitivity of *C. siamense* to multiple types of fungicides.

Does loss of the *CsSCS7* gene affect the plasma membrane permeability and modulate the expression of drug efflux pump-related genes, thus altering fungicide susceptibility of *C. siamense*? We detect the plasma membrane permeability by Evan’s blue (24). After 5 min of staining, spectrophotometry quantitation has showed that the mycelia of the Δ*CsSCS7* strain exhibit a higher stain content than HN08 and ∆*CsSCS7/CsSCS7*. The relative contents of Evan’s blue of HN08, Δ*CsSCS7*, and ∆*CsSCS7/CsSCS7* strains were 0.12 mg/mL, 0.14 mg/mL, and 0.53 mg/mL, respectively. Compared with the relative fluidity of HN08, the relative fluidity of Δ*CsSCS7* and ∆*CsSCS7/CsSCS7* was 23.3% and 89.2%, respectively. (Fig. 4D). Then, we evaluated drug efflux pump-related genes *ZEB2*, *ABCA3*, *PMd1*, *hxnp-1*, and *mfsd1* in *ΔCsSCS7*, ∆*CsSCS7/CsSCS7*, and HN08 by quantitative real-time PCR (qRT-PCR) (25–27). The results showed that the expression of these genes has all decreased in *ΔCsSCS7* (Fig. 4E). These data showed that the loss of *CsSCS7* gene affects the plasma membrane permeability and changes the expression level of drug efflux pump-related genes, which may be the reason for Δ*CsSCS7* fungicide sensitivity increasing.

### The protein domains of csscs7 are required for *C. siamense* hyphal growth

**Fig 5:**
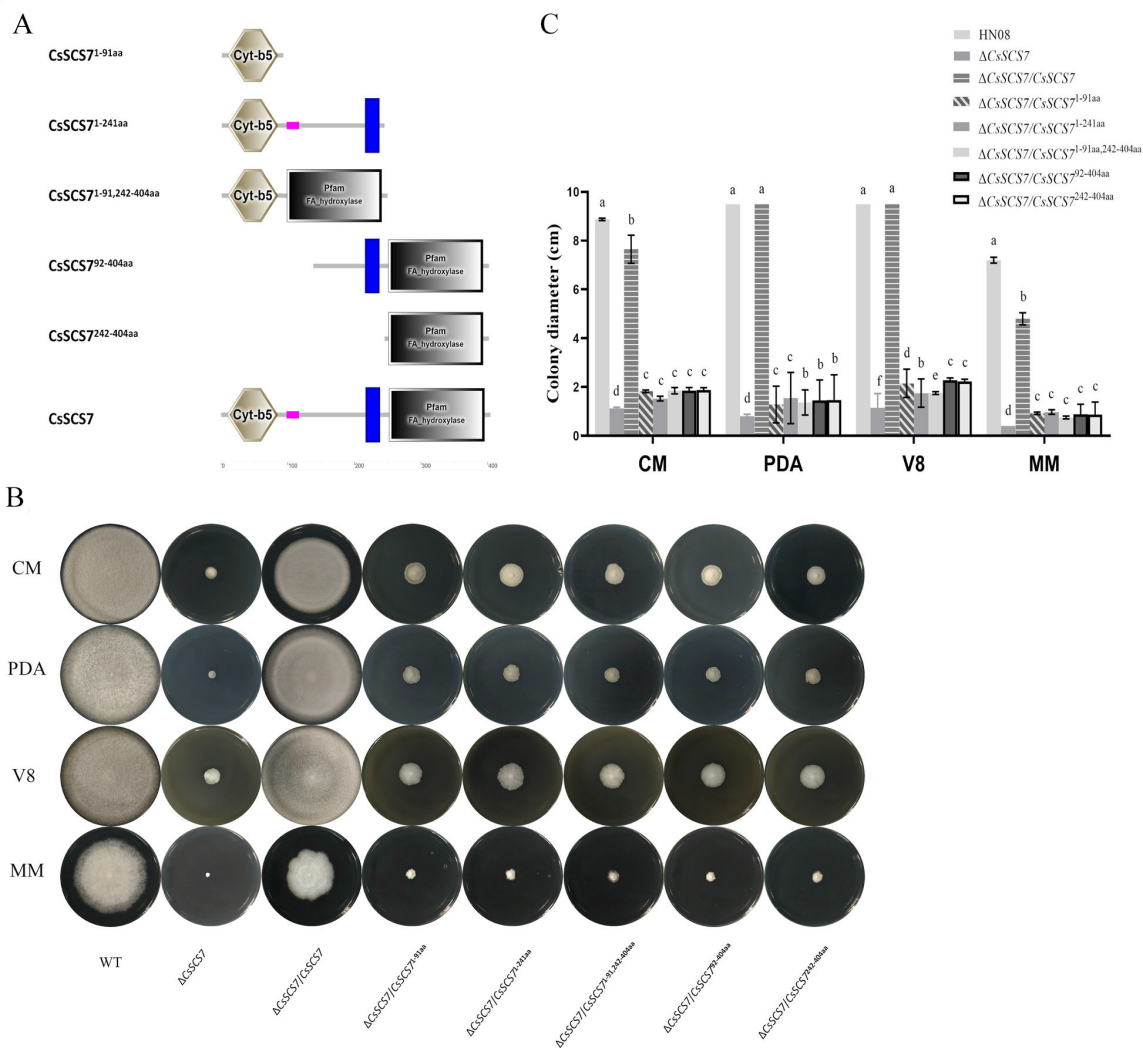
Schematic diagram, colony morphology, and colony diameter of transformants expressing different protein domains. (**A**) Schematic diagram of the structures of the sequences containing different domains of the CsSCS7 protein. (**B**) Colony morphology and (**C**) diameter of transformants expressing the different protein domains grown on four types of plates for 7 days. Different letters indicate significant differences at *P* < 0.01 according to one-way ANOVA and Duncan’s test; the error bars show the standard deviations.

To identify the role of protein domains in the CsSCS7 protein, we reintroduced various parts of *CsSCS7* gene sequences with different domains into Δ*CsSCS7* and constructed serious transformants with CsSCS7 ^1-91 aa^ (Cyt b5 domain), CsSCS7 ^1-241 aa^ (Cyt b5 domain and transmembrane domain), CsSCS7 ^1-91,242-404 aa^ (Cyt b5 domain and hydroxylase domain), CsSCS7 ^92-404 aa^ (transmembrane domain and hydroxylase domain), and CsSCS7 ^242-404 aa^ (hydroxylase domain). The colony diameters of the serious transformants after growth on CM, PDA, V8, and MM plates for 7 days at 28°C were compared (Fig. 5). The transformants containing partial sequences of the *CsSCS7* gene could not restore the colony growth of the Δ*CsSCS7* mutant with the exception of Δ*CsSCS7/CsSCS7* with the whole coding sequence. These results indicated that all three protein domains are required for the role of the CsSCS7 protein in *C. siamense* hyphal growth.

### The *CsSCS7* gene is important for balancing the contents of fatty acids in *C. siamense*

**Fig 6:**
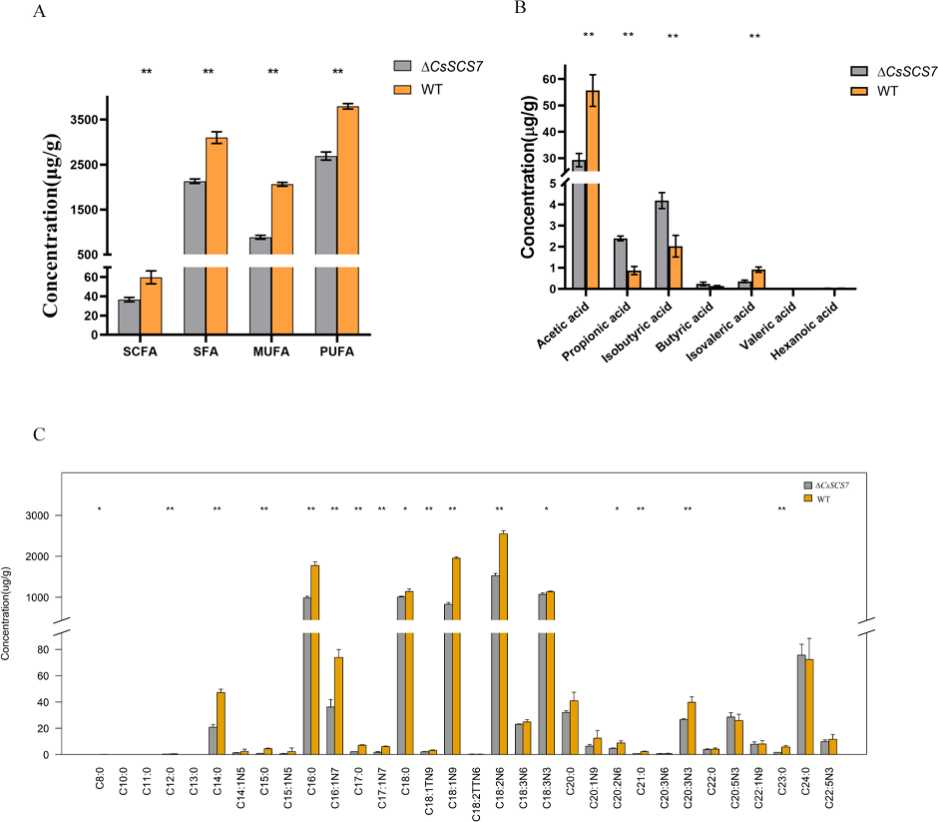
Content of fatty acids in the wild-type strain and ∆*CsSCS7*. (**A**) Total content of four types of fatty acids in the wild-type and ∆*CsSCS7* strains. SCFAs, short-chain fatty acids; SFAs, saturated fatty acids; MUFAs, monounsaturated fatty acids; PUFAs, polyunsaturated fatty acids. (**B**) Content of seven short-chain fatty acids in the wild-type strain HN08 and ∆*CsSCS7*. (**C**) Content of 32 medium- and long-chain fatty acids in the wild-type strain HN08 and ∆*CsSCS7*. The asterisk indicates significant differences within each measurement group (*P < 0.1 and **P < 0.01 according to one-way ANOVA and Duncan’s test). The error bars show the standard deviations.

To evaluate whether *CsSCS7* is involved in the synthesis and metabolism of fatty acids in *C. siamense*, We compared the content of seven short-chain fatty acids and 40 mid-long-chain fatty acids in the wild-type strain HN08 and Δ*CsSCS7* using gas chromatography-mass spectrometry (GC-MS) technology (Fig. 6; Table S3). The results showed that the total content of fatty acids in Δ*CsSCS7* was significantly lower than that in wild-type HN08. Among the 7 short-chain fatty acids, the contents of acetic acid and isovaleric acid among the short-chain fatty acids were significantly lower in the mutant Δ*CsSCS7*, but the contents of propionic acid and isobutyric acid in Δ*CsSCS7* were significantly higher than those in the wild-type strain. No significant difference in the content of the other three short-chain fatty acids (butyric acid, hexanoic acid, and valeric acid) was found between Δ*CsSCS7* and HN08. Forty mid-long-chain fatty acids were tested, and 32 of these mid-long-chain fatty acids were detected successfully. Among these, the content of the following 17 mid-long-chain fatty acids was significantly reduced in Δ*CsSCS7* compared with the wild-type strain: methyl linoleate, methyl oleate, methyl palmitate, methyl stearate, methyl linolenate, methyl palmitoleate, methyl elaidate, methyl heptadecanoate, methyl cis-10-heptadecenoate, methyl myristate, methyl pentadecanoate, methyl dodecanoate, methyl heneicosanoate, methyl tricosanoate, cis-11,14,17-eicosatrienoic acid methyl ester, cis-11,14-eicosadienoic acid methyl ester, and methyl octanoate. These data showed that the loss of the *CsSCS7* gene decreased the content of acetic acid and isovalerate, increased the accumulation of isobutyric acid and propionic acid, and affected the content of 17 mid-long-chain fatty acids, indicating that the *CsSCS7* gene is important for balancing the contents of multiple fatty acids in *C. siamense*.

### The role of SCS7 homologs in hyphal growth may be conserved in filamentous fungi

SCS7 is not essential for *S. cerevisiae* viability (19), but its homolog CsSCS7 is a key gene for *C siamense* hyphal growth. Whether the SCS7 homolog is an important factor for hyphal growth in filamentous fungi remains unknown. We cloned the homologous coding genes from *M. oryzae* (*MoSCS7*, XP_003719946.1), *F. graminearum* (*FgSCS7*, XP_01132368.1), and *S. cerevisiae* (*ScSCS7*, NP_013999.1), linked them with the pXY203 vector, and then reintroduced these plasmids into the *∆CsSCS7* mutant, yielding the complementation strains Δ*CsSCS7/MoSCS7*, Δ*CsSCS7/FgSCS7*, and Δ*CsSCS7/ScSCS7*, respectively.

We compared the mycelial growth of wild-type HN08, Δ*CsSCS7*, Δ*CsSCS7/CsSCS7*, Δ*CsSCS7/MoSCS7*, Δ*CsSCS7/FgSCS7*, and Δ*CsSCS7/ScSCS7* on four types of plates for 7 days at 28°C (Fig. 2A and B; Table S2). The results showed that *MoSCS7* from *M. oryzae* could mostly recover the colony diameter growth rate of the Δ*CsSCS7* mutant, and *FgSCS7* but not *ScSCS7* from *S. cerevisiae* could partially recover the colony diameter growth rate of the Δ*CsSCS7* mutant (Fig. 2A and B). The conidial sizes and spore germination rate of the transformants were also measured (Fig. 2C through E; Table S3), and the results showed that the conidial sizes and spore germination rates of the ∆*CsSCS7* mutant were recovered by *MoSCS7* and *FgSCS7* but not *ScSCS7*. Furthermore, the virulence of the Δ*CsSCS7* mutant can also be recovered by reintroducing the sequences of the *MoSCS7*, *FgSCS7*, and *ScSCS7* genes (Fig. 3C and D). These data indicated that the function of *SCS7* homologs in the hyphal growth of filamentous fungi was conserved.

### The spraying of *C. siamense* with dsRNAs targeting the *csSCS7* gene can reduce mycelial growth and spore germination and attenuates the virulence

**Fig 7:**
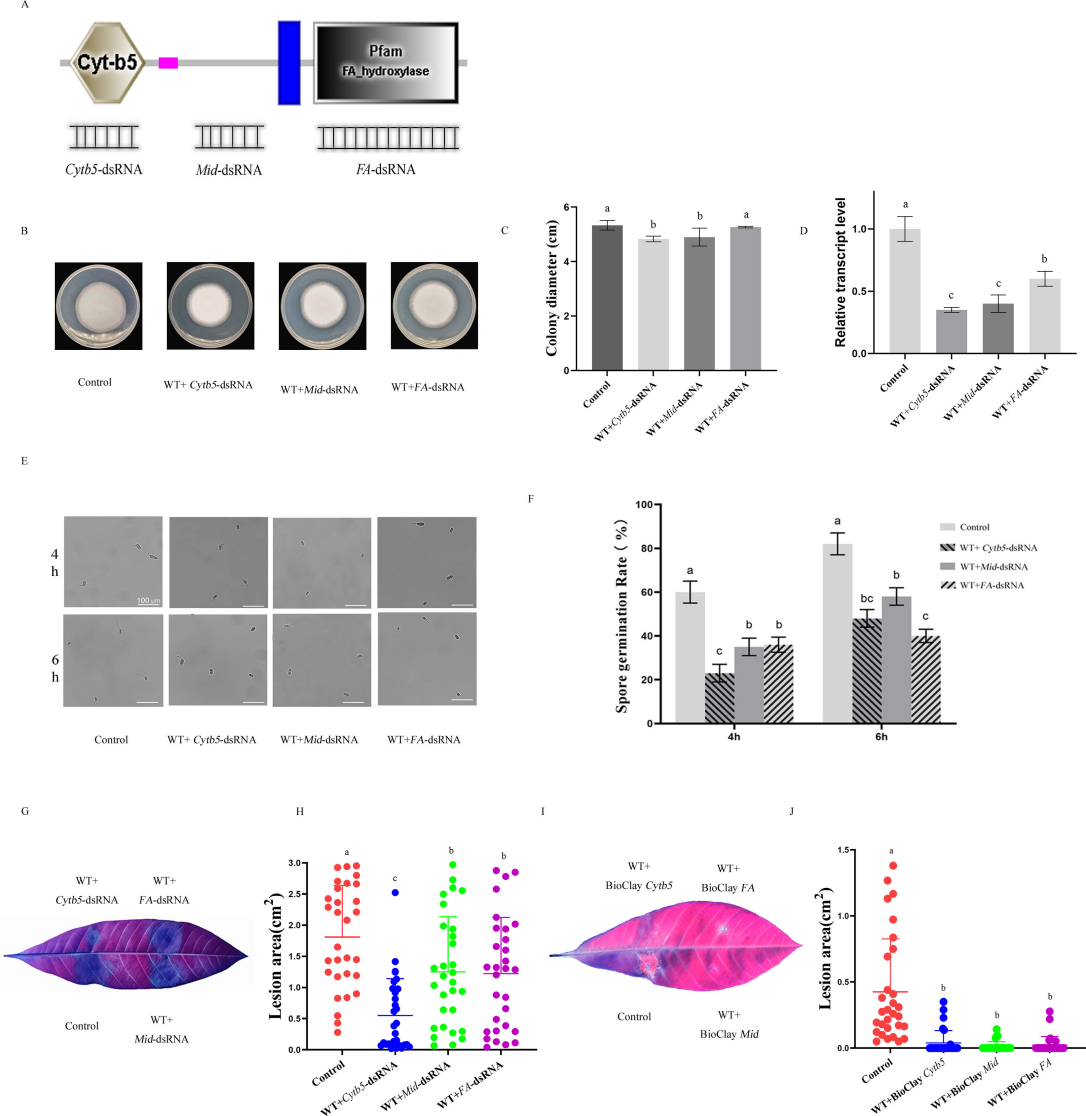
Effects of dsRNAs targeting *CsSCS7* gene on *C. siamense* growth and virulence. (**A**) Schematic diagram of dsRNAs targeting different regions of the *CsSCS7* gene. (**B**) Colony morphology and (**C**) colony diameter of HN08 coinoculated or not coinoculated with *Cytb5*-dsRNA, *Mid*-dsRNA, and *FA*-dsRNA and grown on CM plates for 4 days. (**D**) Expression levels of *CsSCS7* in *C. siamense* HN08 grown on CM plates 4 days after spraying with dsRNAs. (**E**) Germinating spores and (F) spore germination rate of HN08 coinoculated or not coinoculated with three dsRNAs for 4 and 6 h. (**G**) Disease lesions and (**H**) lesion area of rubber tree leaves caused by *C. siamense* conidia coinoculated or not coinoculated with three dsRNAs. (**I**) Disease lesions and (**J**) lesion area of rubber tree leaves caused by *C. siamense* conidia coinoculated or not coinoculated with three BioClay. Different letters indicate significant differences at *P* < 0.01 according to one-way ANOVA and Duncan’s test; the error bars show the standard deviations.

The spraying of dsRNA for induced-gene silencing (SIGS) is an effective way to protect crops both before and after harvest against fungal pathogens (28). We found that fluorescein-labeled *eGFP*-dsRNA could be absorbed by *C. siamense* (Fig. S2). Because *CsSCS7* is an important growth factor for *C. siamense*, we used the SIGS technology to evaluate whether *CsSCS7* could be used as a target in disease control and prevention. Three dsRNAs targeting the Cyt b5 domain (*Cytb5*-dsRNA), hydroxylase domain (*FA*-dsRNA), and intermediate sequence (*Mid*-dsRNA) of the *CsSCS7* gene were designed and synthesized (Fig. 7A). Three types of dsRNA were individually coincubated with conidia on CM culture. The results showed that the coincubation of most *C. siamense* conidia with different dsRNAs slightly decreased the colony growth rate and conidia germination rate. In addition to *FA*-dsRNA, the relative growth rate of *C. siamense* HN08 coincubated with *Cytb5*-dsRNA and *Mid*-dsRNA was on average 90.62% and 91.87% lower than that of HN08 at 4 days after treatment, respectively (Fig. 7B and C). We performed qRT-qPCR and found that the *CsSCS7* gene in *C. siamense* mycelia under three individual dsRNA coincubation conditions was significantly downregulated after 4 days of treatment. Regarding the conidia germination rate, the effect of *C. siamense* conidia germination was decreased by coincubation with *Cytb5*-dsRNA, *Mid*-dsRNA, and *FA*-dsRNA individually. At 4 hai, the conidial germination rate of HN08 coincubated with *Cytb5*-dsRNA, *Mid*-dsRNA, and *FA*-dsRNA averaged 23.42%, 35.36%, and 36.73%, respectively, whereas that of the control was 59.78%. The same results were observed at 6 hai (Fig. 7E and F ). Conidial suspensions (approximately 10^4^/mL) of the wild-type HN08 strain incubated or not incubated with dsRNAs were dropped onto healthy leaves. We found that rubber tree leaves treated with each of the three dsRNAs produced smaller diseased areas compared with the control; specifically, the lesion areas obtained with *Cytb5*-dsRNA, *Mid*-dsRNA, and *FA*-dsRNA were reduced by 69.62%, 19.95%, and 24.16%, respectively. Among these, *Cytb5*-dsRNA demonstrated the best control effect on disease lesions. And then, the nano material Mg-Al layered double hydroxide (LDH) was used as carriers to improve the control effect of dsRNA. We used LDH as carriers to deliver biologically active dsRNA, a formulation termed BioClay (29). The results showed LDH enhanced the control effect of dsRNA significantly, and the lesion areas were obviously reduced, and the lesion areas obtained with BioClay*-Cytb5*, BioClay*-Mid*, and BioClay*-FA* were reduced by 76.47%, 80.86%, and 76.60%, respectively. These data indicate that *CsSCS7* may be a potential control target in *C. siamense*, and nano material LDH can enhance the control effect.

## DISCUSSION

The key genes for the growth and virulence of a pathogen may be used as targets for plant disease control. SCS7 was previously reported as a ceramide very long-chain fatty acid hydroxylase in *S. cerevisiae* that is not essential for yeast growth. In this study, we cloned the SCS7 homolog gene *CsSCS7* from *C. siamense* and characterized its functions in filamentous fungi. We found that *CsSCS7* is involved in the synthesis and metabolism of fatty acids and is vital to the growth and virulence of *C. siamense*. Homologs from *M. oryzae* and *F. graminearum* can recover the retarded hyphal growth obtained with *CsSCS7* gene deletion. All protein domains are important to protein function. We also found that *CsSCS7* plays an important role in balancing the fatty acid content of *C. siamense* mycelia. Furthermore, we analyzed the effect of spraying with dsRNAs targeting *CsSCS7*, and the results showed that this treatment can downregulate *CsSCS7* gene expression and influence the growth and virulence of *C. siamense* to some extent. Then, we used nano material LDH as carriers to deliver dsRNA; the inhibition rates were significantly increased. Taken together, the findings suggest that *CsSCS7* and its homologs are involved in hyphal growth and virulence and may be a potential target in the control of *C. siamense* and even filamentous fungi. This study will provide further insights into the role of fatty acid hydroxylases in fungal growth and virulence and underscore the potential of using CsSCS7 as a potent target in the control of plant pathogenic filamentous fungi.

Fungal sphingolipids are essential constituents of cell membranes and play a pivotal role in growth, morphogenesis, and virulence. Sphingolipids are composed of a sphingoid base or long-chain base amide linked to a fatty acid, which forms the ceramide backbone (5, 30). Hydroxylation of the sphingoid base can add structural diversity that appears to be important for the physiological function of sphingolipids (4). In yeast, ceramide can be classified into five types (Cer-A, Cer-B, Cer-B′, Cer-C, and Cer-D) according to the state of fatty acid hydroxylation. Sur2p and Scs7p are two vital fatty acid hydroxylases in the ceramide synthesis pathway of *S. cerevisiae* (31). Sur2p can catalyze the C-4 hydroxylation of dihydrosphingosine into phytosphingosine, which is then converted to Cer-B by N-acylation (31). Scs7p catalyzes the hydroxylation of the C-2 position fatty acid in Cer-A and Cer-B, yielding Cer-B′ and Cer-C, respectively (19, 31). Neither Scs7 nor Sur2 are essential for yeast (nonfilamentous fungus) growth (19). However, yeasts are unicellular organisms, and studies on the functions of fatty acid hydroxylase in filamentous fungi are limited. It has been reported that the Sur2 homolog BasA in *Aspergillus nidulans* is not only required for phytosphingosine biosynthesis but also essential for hyphal growth and normal sporulation patterns (20). The function of fatty acid hydroxylase SCS7 homologs in filamentous fungi has not been reported. This study demonstrates that *C. siamense* CsSCS7 is also required for hyphal growth and sporulation. Homologs from *M. oryzae* and *F. graminearum* can recover hyphal growth defects induced by *CsSCS7* gene deletion, implying that the function of SCS7 orthologs in hyphal growth is conserved in filamentous fungi. It has also been reported that *basA* in *A. nidulans is* involved in regulating the sphingolipid complex balance between sexual and asexual morphological differentiation in filamentous fungi. Unlike unicellular yeasts, hyphae have a typical filamentous cell morphology. We therefore hypothesized that genes related to fatty acid hydroxylase, such as *CsSCS7* and *basA*, may play an important role in the formation of hyphae in filamentous fungi. Taken together, these results imply that sphingolipid hydroxylation is important for hyphal growth and sporulation in filamentous fungi.

The *S. cerevisiae* SCS7 mutant is resistant to syringomycin E and the antitumor agent PM02734 (32). Orthologs of SCS7 in mammals (FA2H) (17, 33), plants (FAH1, FAH2), and protists have also been characterized (4, 34, 35). The *FA2H* gene in mammals is involved in drug resistance and is related to nervous system diseases (17, 32, 33). FAH1 and FAH2 in *Arabidopsis thaliana* and other plants have been identified and confirmed to be necessary for the organization of plasma membrane nanodomains and associated with resistance to oxidative stress or disease stress (23, 34–38). Here, we also found that *CsSCS7* disruption disturbed the plasma membrane permeability and the expression of drug efflux pump-related genes; the *CsSCS7* deletion mutant showed more sensitivity to multi fungicides. Sphingolipids are also involved in a variety of signal transduction processes and stress responses in multiple organisms (30, 39–45). It is clear that sphingolipids can serve as signaling molecules that contribute to azole fungicide resistance through modulation of the expression of drug efflux pumps (30). *S. cerevisiae SCS7* mutants show reductions in the complex sphingolipid contents and the accumulation of ceramide (31). Our results showed that deletion of the *CsSCS7* gene resulted in a sharp reduction in the total fatty acid content. We hypothesized that the deletion of CsSCS7 affects fungicide resistance by changing the components of the plasma membrane sphingolipids, causing lipid raft deformity and modulating the expression of drug efflux pumps.

In recent years, spray-induced gene silencing has become an innovative and environmentally friendly technology in which the topical application of pathogen gene-targeting dsRNAs or sRNAs molecules onto plant material can enable disease control. To date, SIGS has been used effectively to control a wide range of insect pests, viruses, and pathogenic fungi (46–49). However, SIGS for disease control is dependent on the efficiency of pathogen RNA uptake (28). Many aggressive fungal pathogens can take up RNAs from the environment (28, 50–52), and examples include *Botrytis cinerea*, *Fusarium graminearum*, *Sclerotinia sclerotiorum*, *Fusarium asiaticum*, *Fusarium oxysporum*, *Mycosphaerella fijiensis*, *Aspergillus niger*, and *Rhizoctonia solani* (28, 46, 53, 54). However, Qiao et al. found that *C. gloeosporioides* could not take up environmental dsRNAs (28). In our study, we confirmed that *C. siamense* could take up environmental dsRNAs (Fig. S2). Because CsSCS7 was confirmed as a key factor for hyphal growth and pathogenicity, we believe that it has potential as a control target. Substances inhibit *CsSCS7* gene expression, and small-molecule compounds targeting CsSCS7 may act as inhibitors of filamentous fungi. Our study also demonstrated that spraying with naked *CsSCS7*-dsRNA slightly reduced the inhibition of hyphal growth and disease lesion area, which confirmed that the *CsSCS7* gene can serve as a disease control target. However, the inhibitory effect is not very obvious, and we speculate that this finding may be due to the limited absorption of dsRNAs by *C. siamense*. A method of increasing the durability of dsRNAs may be helpful to improve the disease control effect. And our assays do confirm that using a nanocarrier for dsRNA encapsulation can significantly enhance the disease control of rubber tree anthracnose.

In summary, the SCS7 homolog gene *CsSCS7* in *C. siamense* was functionally analyzed in this study. The *CsSCS7* gene is involved in the synthesis and metabolism of fatty acids and plays a critical role in hyphal growth in *C. siamense* and even in filamentous fungi, and deletion of the *CsSCS7* gene resulted in a sharp reduction in the full virulence and increasing the fungicide sensitivity. Furthermore, we verified that spraying with CsSCS7-dsRNA targeting *CsSCS7* can inhibit hyphal growth and reduce the disease lesion area to some extent. Especially, the inhibition rates were significantly increased after using a nanocarrier. Our study not only provides the demonstration of the function of CsSCS7 in filamentous fungi but also underscores the potential of deploying CsSCS7 and even other fatty acid hydroxylases as potent targets for the control of plant pathogenic filamentous fungi.

## MATERIALS AND METHODS

### Fungal strains and culture conditions

The *C. siamense* HN08 strain was used as a wild-type (WT) strain in this study. The gene deletion mutant Δ*CsSCS7* and the complementary strains Δ*CsSCS7/CsSCS7*, Δ*CsSCS7/MoSCS7*, Δ*CsSCS7/FgSCS7*, Δ*CsSCS7/ScSCS7*, Δ*CsSCS7/CsSCS7 ^1-91 aa^* (Cyt b5 domain), Δ*CsSCS7/CsSCS7 ^1-241 aa^* (Cyt b5 domain and transmembrane domain), Δ*CsSCS7/CsSCS7 ^1-91,242-404 aa^* (Cyt b5 domain and hydroxylase domain), Δ*CsSCS7/CsSCS7 ^92-404 aa^* (transmembrane domain and hydroxylase domain), and Δ*CsSCS7/CsSCS7 ^242-404 aa^* (hydroxylase domain) were constructed from HN08 in this study. For the collection of conidia, we placed and cultured hyphae on PDA medium (20 g/L potato, 20 g/L glucose, and 18 g/L agar) for 3 days and then cultured them under continuous fluorescent light for 3–5 days at 28°C. For the collection of mycelia, the hyphae were cultured in liquid complete medium (CM: 0.6% yeast extract, 0.1% casein hydrolysate, and 1% sucrose) at 28°C with shaking at 150 rpm for 3–5 days.

### *CsSCS7* gene cloning and sequence analysis

The sequence of CsSCS7 was obtained previously from the yeast cDNA library of *C. siamense* HN08 using the CsPbs2 protein of *C. siamense* as the bait protein (22). The *CsSCS7* coding regions and upstream and downstream sequences were obtained from the HN08 genome database and transcriptome database through a local BLAST search. We designed the primers SCS7-F/SCS7-R for amplification of the whole coding sequences of *CsSCS7* DNA and cDNA. The cDNA sequence was transformed into an amino acid sequence and analyzed using Modular Architecture Research Tool (SMART, http://smart.embl-heidelberg.de/, accessed on 15 Oct 2022). The phylogenetic tree was constructed by the maximum-likelihood method using MEGA7 with 1,000 bootstrap values.

### *CsSCS7* gene deletion*,* complementation, and southern blotting

Homologous recombination for *CsSCS7* gene deletion was performed as described previously (21, 55). According to the sequence of *CsSCS7* obtained above, we designed CsSCS7-UF/CsSCS7-UR and CsSCS7-DF/CsSCS7-DR to amplify fragments upstream and downstream of the ORF region and named them U (upstream) and D (downstream), and the linkers were added individually. Using *Xho*I to digest the pCX62-S vector, the U fragment was ligated to the N terminus of the *ILV1* gene in the pCX62-S vector by homologous recombination, and the pCX62-S-U plasmid was obtained. The same method was also used to link the D fragment digested with *Bam*HI to the C terminus of *ILV1* in the pCX62-S-U vector. After confirmation of the sequence, the correct plasmid was named pCX62-S-CsSCS7. The resulting *C. siamense* HN08 protoplasts and transformants were screened as described by Song et al. (56).

To determine the number of sulfonylurea resistance cassettes inserted, we extracted the genomic DNA of the ∆*CsSCS7* and wild-type HN08 strains and digested the obtained DNA with *Eco*RI. The primers S2F/S1R were used to amplify the partial sequence of the *ILV1* gene to be probed. The abovementioned genomic DNA was hybridized with the 616-bp PCR probe and labeled with digoxigenin (DIG)-dUTP using the DIG Hing Prime DNA Labeling and Detection Starter Kit I (Roche, Basel, Switzerland).

For the complement mutant strain, pXY203 (with the hygromycin transferase gene *HPH* and RP27 promoter) was used to construct the gene complementation vector. The pXY203 vector was digested with the restriction endonuclease *Xho*I. Primers with homology arms were used to amplify *MoSCS7*, *FgSCS7*, *ScSCS7*, CsSCS7 ^1-91 aa^ (Cyt b5 domain), CsSCS7 ^1-241 aa^ (Cyt b5 domain and transmembrane domain), CsSCS7 ^1-91,242-404 aa^ (Cyt b5 domain and hydroxylase domain), CsSCS7 ^92-404 aa^ (transmembrane domain and hydroxylase domain), and CsSCS7 ^242-404 aa^ (hydroxylase domain) fragments, which were cotransformed with the linearized pXY203 vector into yeast XK1-25 for yeast homologous recombination. The transformants of the positive clones were extracted and then transferred into *E. coli* DH5α to obtain the correct plasmid. The plasmid was introduced into ∆*CsSCS7* mutant protoplasts, and the transformants were screened by PDS (200 g/L potatoes, 20 g/L dextrose, 274 g/L sucrose, and 20 g/L agar) medium with 600 µg/mL hygromycin and verified by PCR, which yielded the complementary strains *∆CsSCS7/CsSCS7*, *∆CsSCS7/MoSCS7*, *∆CsSCS7/FgSCS7*, *∆CsSCS7/ScSCS7*, Δ*CsSCS7/CsSCS7 ^1-91 aa^* (Cyt b5 domain), Δ*CsSCS7/CsSCS7 ^1-241 aa^* (Cyt b5 domain and transmembrane domain), Δ*CsSCS7/CsSCS7 ^1-91,242-404 aa^* (Cyt b5 domain and hydroxylase domain), Δ*CsSCS7/CsSCS7 ^92-404 aa^* (transmembrane domain and hydroxylase domain), and Δ*CsSCS7/CsSCS7 ^242-404 aa^* (hydroxylase domain).

### Phenotype analysis

Conidia of these strains were collected as described above. Using a hemocytometer, the concentration of the spores was adjusted to 10^5^/mL, and 10 µL of the spore suspensions was dropped onto the center of medium plates. The growth rate of the individual colonies was assessed after 5 dpi under a steady temperature of 28°C. Twenty microliters of spore suspensions (1 × 10^5^ spores/mL) was inoculated onto the surface of microslides, and after incubation at 28°C, the conidia morphology was examined under the microscope. One hundred spores of each strain were tested. Three independent experiments were performed.

The responses of the tested strains to different fungicides were examined using 10 µL of a suspension of conidia (10^5^/mL) from the defective, wild-type, and complementation strains inoculated on CM supplemented with different contents of fungicides and cultured at 28°C. The colony diameter was measured and photographed at 5 dpi.

For the plasma membrane integrity, Evan’s blue was adopted to detect HN08, ∆*CsSCS7*, and ∆*CsSCS7/CsSCS7*. The method was previously described (24). Strains were cultured with liquid medium CM for 3 days and then treated with 0.01% Evan’s blue solution for 15 min. washing strains with 0.1 M CaCl_2_ of PH 5.6. Mycelia were ground into a fine powder in liquid nitrogen and lysed by 1 mL of 1% SDS. The lysates were shaken and centrifugated at 13,000 × *g* for 10 min. The optical density of the supernatants was measured at 600 nm spectrophotometrically (METTLER TOLEDO, Switzerland).

For pathogenicity assessment assays, 10 µL of spore suspensions (1 × 10^5^/mL) of the tested strains was dropped on the tender leaves with or without wounding as described by Wang et al. (21). Three technical replicates of each treatment were included in the experiment, and 30 leaves were inoculated for each treatment. The disease lesions were measured and photographed at 5 dpi.

### Total RNA extraction and qRT-PCR Analysis

Total RNA extraction and qRT-PCR analysis were performed as described by Guan et al. (55). Total RNA of the mycelia of the HN08 strain and mutants were extracted using the RNAprep Pure Plant Kit (Tiangen, Beijing, China). cDNA synthesis was performed with TransScript One-Step gDNA Removal and cDNA Synthesis SuperMix (TransGen Biotech, Beijing, China). The expression levels of the target genes were quantified by qRT-PCR performed with an ABI7500 sequence detection system (Applied Biosystems, Waltham, MA, USA). Reactions were performed in a total volume of 10 µL using the SYBR Premix Dimer Eraser Kit (Takara, Beijing, China). All of the reactions were repeated in at least three independent pools in three sets of biological replicates. The primer sequences were listed in Table S1.

### Determination of the fatty acid content

The measurement of the fatty acid content was entrusted to Shanghai Zhongke New Life Biotechnology Co. Ltd. (Shanghai, China). The standards were mixed into a total of 10 mixed standard concentration gradients, and 500 µL of the standard mixture was collected for GC-MS detection. The volume of each injection was 1 µL, and the split ratio was 10:1 with split injection. The samples were thawed on ice. Then, 100 mg of the thawed samples was placed in 2-mL glass centrifuge tubes on ice, and 1 mL of chloroform-methanol solution was added to the centrifuge tube. After sonication for 30 min, the supernatant was removed and placed in a new syringe. Next, 2 mL of 1% sulfuric acid-methanol solution was added, and the tube was placed in a water bath at 80°C. Subsequently, 1 mL of n-hexane extraction was added to the centrifuge tube, and 5 mL of water was added for washing. For GC-MS, 500 µL of the supernatant was collected from the samples, and 25 µL of methyl salicylate was added as a standard. The mixture was mixed well, transferred to the injection vial, and subjected to GC-MS detection. MSD ChemStation was used to extract the chromatographic peak area and retention time. The amount of long-chain fatty acids in the samples was calculated.

### Application of spray-induced gene silencing technology

Using the Vazyme T7 RNAi Transcription Kit (Nanjing Vazyme Biotech Co. Ltd., CN) according to the instructions, we generated dsRNAs *in vitro*. The RNAi fragments introduced the T7 promoter sequence at the 5′ ends by PCR. After purification, the DNA fragments containing the T7 promoter at the 5′ ends were used for *in vitro* transcription. The primers used for the synthesis of dsRNAs are listed in Table S1.

To determine whether *C. siamense* could take up dsRNAs *in vitro*, fluorescein-labeled *eGFP-*dsRNA was generated using fluorescein-12-dUTP (Thermo Fisher Scientific, Waltham, MA, USA). For microscopy determination of fluorescent dsRNA uptake by conidia of *C. siamense*, 10 µL of 200 ng/µL fluorescent dsRNA was applied to 20 µL of 10^5^ spores/mL. The conidia of *C. siamense* were placed in a 1.5-mL tube, and 20 µL of 200 ng/µL fluorescent dsRNAs was then added. The samples were cultured at 28°C in darkness and coincubated for 24 h before imaging. As the control, 20 µL of H_2_O or 20 µL of 200 ng/µL *eGFP*-dsRNA was added to 20 µL of 10^5^ spores/mL, and the mixture was cultured under the same conditions as those used for the sample. Before observation, spores and mycelium were treated with micrococcal nuclease enzyme (Thermo Fisher Scientific, Waltham, MA, USA) to degrade the dsRNAs, which was on the surface of spores and mycelium, at 37°C for 30 min.

The dsRNAs were used to assess the colony growth, spore germination, and fungal pathogenicity of *C. siamense*. We designed primers for and synthesized three dsRNAs. The dsRNAs were adjusted to a concentration of 200 ng/µL with RNase-free water before use. Conidia were collected as described above, and the concentration of the spores was adjusted to 10^3^/mL. The conidia were placed in a 1.5-mL tube, and 10 µL of 200 ng/µL dsRNAs was added. The samples were then dropped onto the center of plates. The growth rate of the individual colonies was assessed after 4 dpi under a steady temperature of 28°C in darkness. For microscopy examination of dsRNA uptake by spores, 10 µL of 10^5^ spores/mL and 10 µL of 200 ng/µL dsRNAs were coincubated onto the surface of microslides.

For nano material LDH, we examined the concentration of nano materials that can fully load dsRNA. We tested the optimal loading ratio of LDH and dsRNA with LDH concentration of 50 µg/mL and showed that the effective loading radio of LDH and dsRNA is 20 µL:1 µg. And three 200-ng/µL dsRNAs were loaded on LDH. Pathogenicity assessment assays were performed as described above with the exception that 10 µL of 200-ng/µL dsRNAs or 10 µL of 200-ng/µL BioClay was added to the treated area 2 dpi later.

## Data Availability

The data that support the findings of this study are available in the supplemental material of this article and also are available from the corresponding author upon reasonable request.

## SUPPLEMENTAL MATERIAL

Fig. S1 mbio.02015-23-s0001.doc Verification of the gene deletion mutant ΔCsSCS7 and complemented transformant ΔCsSCS7/CsSCS7.

Fig. S2 mbio.02015-23-s0002.doc Fluorescein-labeled eGFP-dsRNA could be absorbed by *C. siamense*.

Table S1 mbio.02015-23-s0003.docx Primer sequences in the study.

Table S2 mbio.02015-23-s0004.docx Colony diameter of the six tested strains in this study.

Table S3 mbio.02015-23-s0005.docx Size of conidia and spore germination rate of the six tested strains in this study.

Table S4 mbio.02015-23-s0006.docx The content of 7 short-chain fatty acid and 32 medium and long-chain fatty acids in wild-type strains HN08 and ΔCsSCS7.

ASM does not own the copyrights to Supplemental Material that may be linked to, or accessed through, an article. The authors have granted ASM a non-exclusive, world-wide license to publish the Supplemental Material files. Please contact the corresponding author directly for reuse.
