# Supplementary material for: The fatty acid 2-hydroxylase CsSCS7 is a key hyphal growth factor and potential control target in Colletotrichum siamense
Source: mBio. 2024 Jan 10;15(2):e02015-23. doi: 10.1128/mbio.02015-23 (PMC10865788; doi:10.1128/mbio.02015-23)
Supplement: Fig. S1 — Verification of the gene deletion mutant ΔCsSCS7 and complemented transformant ΔCsSCS7/CsSCS7. [file mbio.02015-23-s0001.doc]

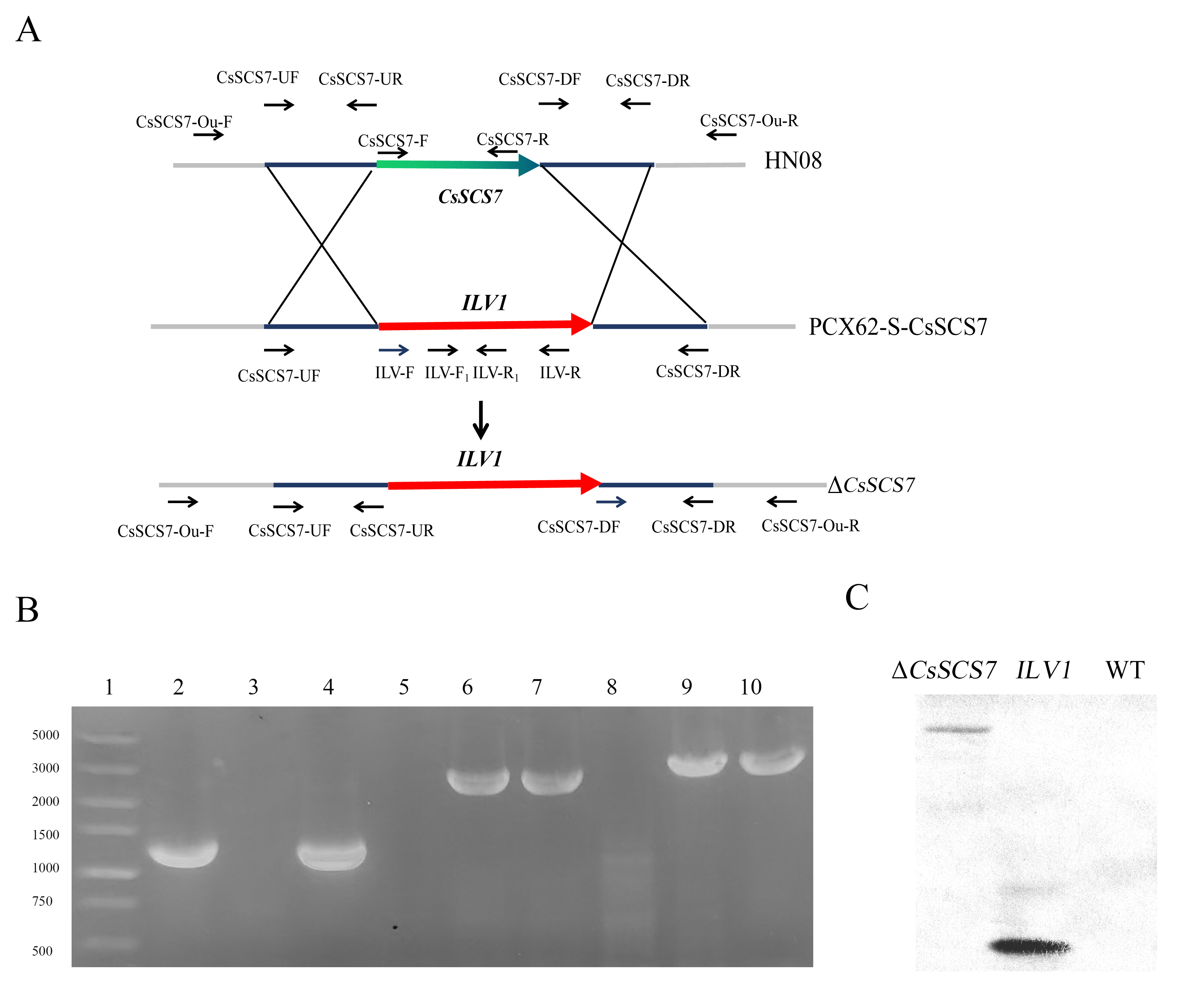
 **Fig. S1** Verification of the gene deletion mutant Δ*CsSCS7* and complemented transformant Δ*CsSCS7/CsSCS7*. (A) Schematic diagram of the construction of the *CsSCS7* gene deletion mutant and primers. (B) PCR verification of the *CsSCS7* gene deletion mutant strain and complemented strain. The internal sequence of the *CsSCS7* gene could be amplified by the primers CsSCS7-F/CsSCS7-R in the wild-type (Lane 2) and Δ*CsSCS7/CsSCS7* (Lane 4) strains but not in the Δ*CsSCS7* strain (lane 3). The sequence with the upstream sequence of *CsSCS7* and partial *ILV1* gene sequence amplified by CsSCS7-Ou-F/ILV-R1 and the fragment with the downstream sequence of *CsSCS7* and partial *ILV1* gene sequence amplified by ILV- F1/CsSCS7-Ou-R are shown in Δ*CsSCS7* (lanes 6 and 9) and Δ*CsSCS7/CsSCS7* (lanes 7 and 10) but not the wild-type (lanes 5 and 8). Lane 1 is the DNA DL5000 marker. (C) Southern blot analysis confirming the *ILV1* gene number in Δ*CsSCS7*. Genomic DNA of the Δ*CsSCS7* mutant (lane 1) and wild-type (lane 3) strains was digested with *Eco*RI and probed with an *ILV1* coding sequence. Lane 2 shows a PCR fragment of *ILV1* as a positive control.
