# Supplementary material for: The fatty acid 2-hydroxylase CsSCS7 is a key hyphal growth factor and potential control target in Colletotrichum siamense
Source: mBio. 2024 Jan 10;15(2):e02015-23. doi: 10.1128/mbio.02015-23 (PMC10865788; doi:10.1128/mbio.02015-23)
Supplement: Fig. S2 — Fluorescein-labeled eGFP-dsRNA could be absorbed by C. siamense. [file mbio.02015-23-s0002.doc]

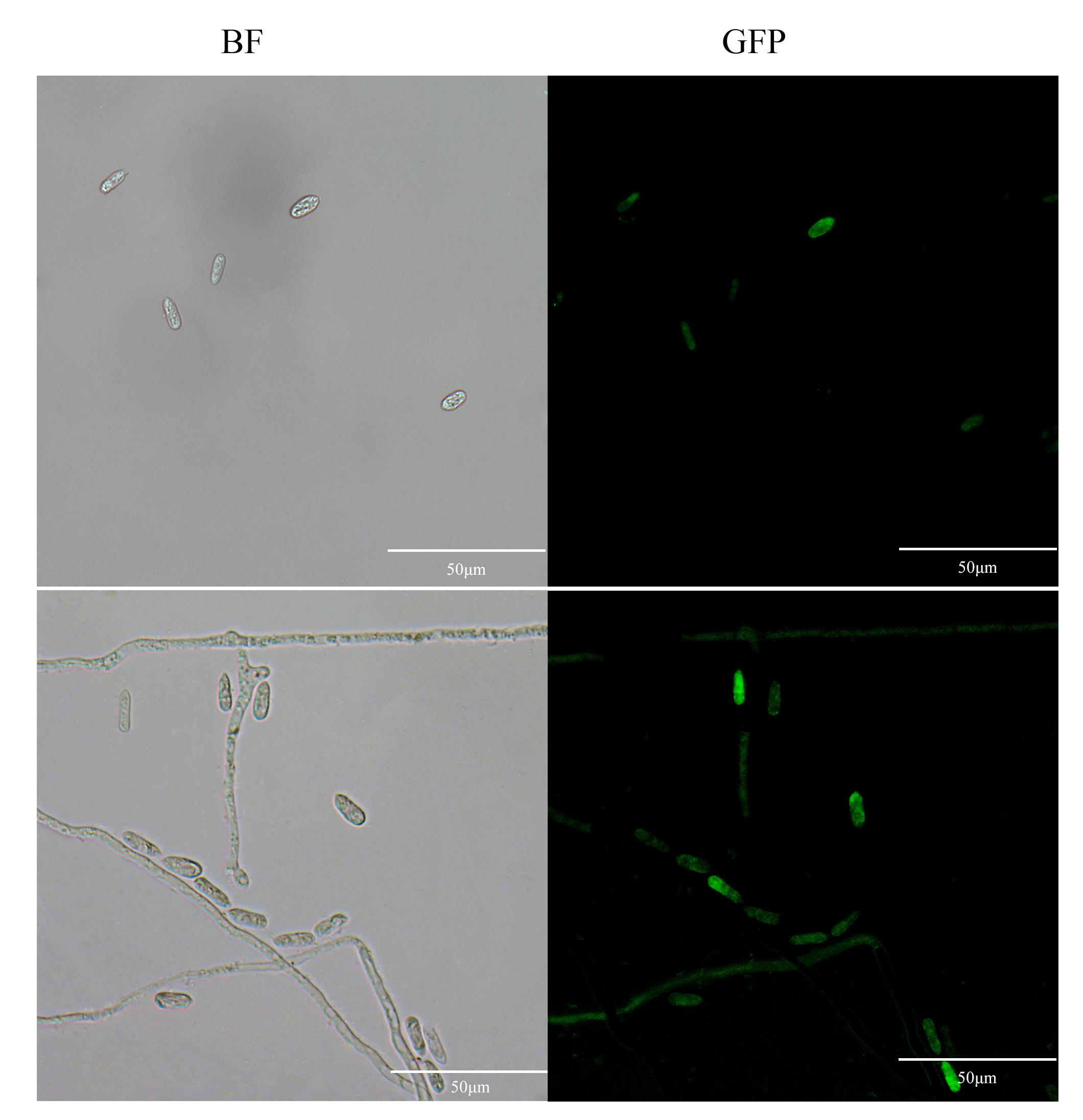


**Fig. S2** Fluorescein-labeled *eGFP*-dsRNA could be absorbed by *C. siamense.*

Fluorescein-labeled *eGFP*-dsRNA was added to spores to determine RNA uptake. Spores of *C. siamense were* placed in a 1.5-mL tube, fluorescent dsRNA was added, and the samples were cultured at 28 °C in darkness and coincubated for 24 h before imaging. MNase treatment was performed at 37 °C for 30 min. BF is bright-field mocroscopy image and GFP is green fluorescence image; Scale bars, 50 µm.
