# Supplementary material for: The fatty acid 2-hydroxylase CsSCS7 is a key hyphal growth factor and potential control target in Colletotrichum siamense
Source: mBio. 2024 Jan 10;15(2):e02015-23. doi: 10.1128/mbio.02015-23 (PMC10865788; doi:10.1128/mbio.02015-23)
Supplement: Table S1 — Primer sequences in the study. [file mbio.02015-23-s0003.docx]

**Table S1 Primer sequences in the study**

| **Primer** | **Sequence 5′-3′** |
| --- | --- |
| CsSCS7-F | CGGAATTCATGCCGTCGAGAACTCTCCCTACCTTC |
| CsSCS7-R | CGGGATCCTTATTGCGTCTTGACAATGGGAGGC |
| CsSCS7-RT-F | ATTTGGCTAGAGAGGGCATGGACAG |
| CsSCS7-RT-R | TGACGAGGCGATACTTGTCCATAGG |
| CsSCS7-UF | GTACCGGGCCCCCCCAGCTTCTCAGAATCCACATATCCAC |
| CsSCS7-UR | CGATACCGTCGACCTCGAAGCTGCAGGTGGCGATCGTGAAT |
| CsSCS7-DF | GCTCTCACCGCGGATCCTTCATCGACCAACGTTCATAC |
| CsSCS7-DR | CTAGAACTAGTGGATCTCTAGGCCGGACATGTTGACTG |
| CsSCS7-OU-F | TGCTTTTGCATTGCTGTGACC |
| CsSCS7-OU-R | ATGCCTTGTGTTCTTCCGGT |
| CsSCS7-OF | TTTCGTAGGAACCCAATCTTCAAAATGCCGTCGAGAACTCTCCCTACCTTC |
| CsSCS7-OR | TTCGAATTTAGCAGCAGCGGTTTCTTTTTGCGTCTTGACAATGGGAGGC |
| ILV-F | GTGCCAACGCCACAGTGCCCCACA |
| ILV-R | GTGAGAGCATGCAATTCCCGTGCAATA |
| ILV-F_1_ | GGCGGTGCTATCCTTCCCGTGTT |
| ILV-R_1_ | GTTCAACGCCGCCTTCCGACAAAAT |
| CsSCS7-1-99aa-F | ATGCCGTCGAGAACT |
| CsSCS7-1-99aa-R | CAAAAGTCAATCAAT |
| CsSCS7-1-241aa-R | ATTGATTGACTTTTG |
| CsSCS7-1-91+242-404aa-F | TTTCGTAGGAACCCAATCTTCAAATGCTCGAGATGCCGTCGAGAACT |
| CsSCS7-1-91+242-404aa-R | CTCATGCTCCTGGGTCCCATTGATTGACTTAAGTCAATCAATGGGACCCAGGAGCATGAG |
| CsSCS7-92-404aa-F | ATGGACAGCACCTTC |
| CsSCS7-92-404aa-R | TTATTGCGTCTTGAC |
| CsSCS7-242-404aa-F | GCCAACAAGCCCAAC |
| Stag-conR | TACGTGCGCAGAGGAGCCTGAAT |
| MoSCS7-F | ATGCCGTCGCTCACACTCCCAT |
| MoSCS7-R | TTGACAAGAGGCACAATCTCGG |
| MoSCS7-RT-F | CAAGCTCAACCTTGCAGCGCTCT |
| MoSCS7-RT-R | AGAATGGGAACGCCAGGACGAAG |
| FgSCS7-F | ATGCCTGGACTTACACTTCC |
| FgSCS7-R | TTTCGTCTTCTTGGTGTCGT |
| FgSCS7-RT-F | ACCAGCTCTACACTCTTGGC |
| FgSCS7-RT-R | GAATTAGCATACATGGCTGG |
| ScSCS7-F | ATGTCGACTAATACTTCCAA |
| ScSCS7-R | TTCATATTTCATTTTGGACA |
| ScSCS7-RT-F | TATTTGCTCTGCTGCCACTT |
| ScSCS7-RT-R | TTCATTTTGGACAATGGGGCATC |
| ZEB2-F | ATGGCTTTCGCCGGGATGGCGTCCAT |
| ZEB2-R | CTCGGTCTTCTTCTTGCCAAAGAGGC |
| ZEB2-RTF | AAGTCGTCGTGTTGTACGAG |
| ZEB2-RTR | GCCTGAAGGGCCTTGTAGTT |
| ABCA3-F | ATGGCGCTTCTACGCCAGACTTGGA |
| ABCA3-R | TCGCCCAAAACGCCACCAGGACCT |
| ABCA3-RTF | GGCTAACTACAACGGTCTTA |
| ABCA3-RTR | TGGTCGAACACCATTGGAAT |
| PMd1-F | ATGGGCACCTCCCGGGAGAAATTA |
| PMd1-R | TGCTGCAGTCAAAGCTGCGTAGTA |
| PMd1-RTF | TACAGCACCCTGTTTACCCT |
| PMd1-RTR | GTTAGTGACGAAGGCCAGAA |
| hxnp2-F | ATGAAGTCCACCACAGATGTTCGG |
| hxnp2-R | AATGAAGAATCTCCATCTTGGGTC |
| hxnp2-RTF | TTCGGAAGTGCTCTCCTAGC |
| hxnp2-RTR | CACGAAGAGACCTCAAGCTT |
| mfsd-F | ATGGATGCCGGTCTGAAATCCCAAG |
| mfsd-R | GATCAAGTACCGCACGAAAACGGC |
| mfsd-RTF | CTGTCGGTTTACAATACCCT |
| mfsd-RTR | TGGCACGATCATCATCAGCT |
| CsCytb5-dsF1 | TAATACGACTCACTATAGGGTTCACCCGCGCCGAGGTCGA |
| CsCytb5-dsR | TAATACGACTCACTATAGGGAATGACGAATCCAACCAAGG |
| CsMid-dsF | TAATACGACTCACTATAGGGCAATCAATGGGACGGCCAAC |
| CsMid-dsR | TAATACGACTCACTATAGGGTAGCAGACCTCCTG |
| CsFA-dsF | TAATACGACTCACTATAGGGTGGGGGCTCGGCTTCTTTCT |
| CsFA-dsR | TAATACGACTCACTATAGGGTTCGGTGCCGAAAATGCTAT |
