# Supplementary material for: The fatty acid 2-hydroxylase CsSCS7 is a key hyphal growth factor and potential control target in Colletotrichum siamense
Source: mBio. 2024 Jan 10;15(2):e02015-23. doi: 10.1128/mbio.02015-23 (PMC10865788; doi:10.1128/mbio.02015-23)
Supplement: Table S2 — Colony diameter of the six tested strains in this study. [file mbio.02015-23-s0004.docx]

| Strain | CM | | PDA | | V8 | | MM | |
| --- | --- | --- | --- | --- | --- | --- | --- | --- |
|  | Colony Diameter（cm）^b^ | The ratio of colony diameter^c^ | Colony Diameter（cm） | The ratio of colony diameter | Colony Diameter （cm） | The ratio of colony diameter | Colony Diameter（cm） | The ratio of colony diameter |
| WT | 8.6±0.05 a | 100% a | 8.53±1.05 a | 100% a | 8.6±0.15 a | 100% a | 3.56±0.11 a | 100% a |
| Δ*CsSCS7* | 2.5±0.05 e | 29.07% e | 1.93±0.03 e | 22.63% e | 3.01±0.09 d | 35.00% d | 0.85±0.00 e | 23.88% e |
| Δ*CsSCS7/CsSCS7* | 8.3±0.07 b | 96.51% b | 8.38±0.03 ab | 98.24% ab | 8.15±0.35 b | 94.77% b | 2.35±0.00 d | 66.01% d |
| Δ*CsSCS7/MoSCS7* | 8.15±0.15 c | 94.77% c | 8.2±0.05 b | 96.13% b | 8.35±0.05 ab | 97.09% ab | 3.25±0.00 b | 91.13% b |
| Δ*CsSCS7/FgSCS7* | 6.36±0.08 d | 73.95% d | 5.38±0.46 c | 63.07% c | 5.67±0.33 c | 65.93% c | 2.88±0.11 c | 80.90% c |
| Δ*CsSCS7/ScSCS7* | 2.51±0.06 e | 29.19% e | 2.26±0.06 d | 26.49% d | 2.61±0.09 e | 30.35% e | 0.75±0.03 e | 21.07% e |

**Table S2 Colony diameter of the six tested strains in this study***^a^*

*^a^* Data provided in all columns were averages with standard deviations.

*^b^* Diameter of colony at day 7 after incubation on four kinds of plates under 28℃, average of 3 measurements.

*^c^* The ratio of colony diameter =(Colony diameter of the strain/ Colony diameter of HN08)×100%.

*^d^* Means followed by the different letters were significantly different at α=0.01, according by Duncan’s new multiple range test.
