# Supplementary material for: The fatty acid 2-hydroxylase CsSCS7 is a key hyphal growth factor and potential control target in Colletotrichum siamense
Source: mBio. 2024 Jan 10;15(2):e02015-23. doi: 10.1128/mbio.02015-23 (PMC10865788; doi:10.1128/mbio.02015-23)
Supplement: Table S3 — Size of conidia and spore germination rate of the six tested strains in this study. [file mbio.02015-23-s0005.docx]

**Table S3 Conidia size and spore germination rate of the six tested strains in this study**

| Strain | Conidia length（μm）^b^ | Conidia Width（μm） | spore germination rate^a^ | | |
| --- | --- | --- | --- | --- | --- |
|  |  |  | 2h | 4h | 6h |
| WT | 13.36±1.98 a | 4.50±0.78 a | 6.00% a | 82.00% a | 87.00% a |
| Δ*CsSCS7* | 10.91±0.58 d | 3.73±0.19 d | 0.00% d | 26.30% d | 48.00% c |
| Δ*CsSCS7/CsSCS7* | 12.40±0.95 b | 3.93±0.45 bc | 5.67% a | 74.30% b | 84.60% a |
| Δ*CsSCS7/MoSCS7* | 12.26±2.14 ab | 4.05±0.32 b | 4.00% ab | 70.30% bc | 86.00% a |
| Δ*CsSCS7/FgSCS7* | 12.48±1.35 ab | 3.93±0.21 bc | 3.30% bc | 73.00% bc | 81.60% a |
| Δ*CsSCS7/ScSCS7* | 11.44±1.01 c | 3.63±0.35 d | 2.33% c | 66.70% c | 77.30% ab |

*^a^* germination rate of spore, average of 300 measurements.

*^b^*Length and width of conidia, average of 300 measurements.

*^c^* Means followed by the different letters were significantly different at α=0.01, according by Duncan’s new multiple range test.
