# Supplementary material for: The fatty acid 2-hydroxylase CsSCS7 is a key hyphal growth factor and potential control target in Colletotrichum siamense
Source: mBio. 2024 Jan 10;15(2):e02015-23. doi: 10.1128/mbio.02015-23 (PMC10865788; doi:10.1128/mbio.02015-23)
Supplement: Table S4 — The content of 7 short-chain fatty acid and 32 medium and long-chain fatty acids in wild-type strains HN08 and ΔCsSCS7. [file mbio.02015-23-s0006.docx]

**Table S4 The content of 7 short-chain fatty acid and 32 medium and long-chain fatty acids in wild-type strains HN08 and Δ*CsSCS7***

| **Fatty Acids** | **Mean content of HN08 (Unit: µg/g)** | **Mean content of Δ*CsSCS7* (Unit: µg/g)** |
| --- | --- | --- |
| Acetic acid | 55.6480±5.3621a | 29.2921±1.3524b |
| Propionic acid | 0.8741±0.1337a | 2.3965±0.1425b |
| Isobutyric acid | 2.0275±0.4879a | 4.1884±0.3147b |
| Butyric acid | 0.1456±0.0198 | 0.2410±0.0874 |
| Isovaleric acid | 0.9190±0.1078a | 0.3647±0.0517b |
| Valeric acid | 0.0355±0.0014 | 0.0354±0.0017 |
| Hexanoic acid | 0.0325±0.0017 | 0.0312±0.0015 |
| Methyl octanoate | 0.0869±0.0187a | 0.0483±0.0114b |
| Methyl decanoate | 0.0303±0.0056 | 0.0206±0.0041 |
| Methyl undecanoate | 0.0165±0.0049 | 0.0153±0.0039 |
| Methyl dodecanoate | 0.5179±0.0589a | 0.2613±0.0312b |
| Methyl tridecanoate | 0.0275±0.0066 | 0.0178±0.0068 |
| Methyl myristate | 47.2189±1.1352a | 20.8375±0.8871b |
| Methyl myristoleate | 2.3798±1.0121 | 1.3126±0.3317 |
| Methyl pentadecanoate | 4.4575±0.3141a | 0.8155±0.0874b |
| Methyl cis-10-pentadecenoate | 2.2615±1.3141 | 0.7093±0.1555 |
| Methyl palmitate | 1774.4328±88.8213a | 982.8418±44.2145b |
| Methyl palmitoleate | 73.8638±4.4412a | 36.2206±4.1256b |
| Methyl heptadecanoate | 7.0663±0.4111a | 2.2005±0.0879b |
| Methyl cis-10-heptadecenoate | 6.1021±0.2982a | 1.7438±0.3871b |
| Methyl stearate | 1139.3073±31.1241a | 1010.1675±10.3654b |
| Methyl elaidate | 3.1873±0.4123a | 2.1534±0.3564b |
| Methyl oleate | 1953.8933±11.2341a | 828.7013±39.6451b |
| Methyl linolelaidate | 0.1919±0.0901 | 0.2324±0.0678 |
| Methyl linoleate | 2547.6164±60.1456a | 1526.0002±33.1762b |
| Methyl γ-linolenate | 22.9984±0.9124 | 24.8181±1.3142 |
| Methyl linolenate | 1070.3127±26.3124a | 1134.5519±14.3175b |
| Methyl arachidate | 32.0616±1.1276 | 41.0053±3.4173 |
| cis-11-Eicosenoic acid methyl ester | 6.3450±1.0652 | 12.5034±3.1763 |
| cis-11,14-Eicosadienoic acid methyl ester | 4.6732±0.2961a | 8.8823±1.1734b |
| Methyl heneicosanoate | 0.7239±0.0192a | 2.3191±0.5172b |
| cis-8,11,14-Eicosatrienoic acid methyl ester | 0.5931±0.1791 | 0.7078±0.1145 |
| cis-11,14,17-Eicosatrienoic acid methyl ester | 26.6975±0.6173a | 39.7615±2.1453b |
| Methyl behenate | 3.9069±0.3144 | 4.0831±0.5178 |
| cis-5,8,11,14,17-Eicosapentaenoic acid methyl ester | 28.6032±0.2178 | 25.9082±0.4124 |
| Methyl erucate | 7.8006±1.1002 | 8.0619±1.4158 |
| Methyl tricosanoate | 1.6694±0.0217a | 5.7449±0.0881b |
| Methyl tetracosanoate | 75.6119±5.6918 | 72.3489±8.1173 |
| Methyl docosapentaenoate | 9.9318±1.0172 | 11.5844±3.0452 |
| Total_MUFA (monounsaturated fatty acids) | 884.9869±34.8431a | 2062.2534±46.4014b |
| Total_PUFA (polyunsaturated fatty acids) | 2690.0429±61.8251a | 3796.2303±69.5952b |
| Total_SCFA^*^（short chain fatty acids） | 36.5496±1.6872a | 59.6825±5.1149b |
| Total_SFA^*^（saturated fatty acids） | 2131.2004±45.8714a | 3098.6636±112.4617b |

^*^Total_SCFA represents the total content of short-chain fatty acids; Total_SFA represents the total content of mid-long-chain fatty acids.
